# Factorial Model of Obese Adolescents: The Role of Body Image Concerns and Selective Depersonalization—A Pilot Study

**DOI:** 10.3390/ijerph191811501

**Published:** 2022-09-13

**Authors:** Marco La Marra, Antonietta Messina, Ciro Rosario Ilardi, Maria Staiano, Girolamo Di Maio, Giovanni Messina, Rita Polito, Anna Valenzano, Giuseppe Cibelli, Vincenzo Monda, Sergio Chieffi, Alessandro Iavarone, Ines Villano

**Affiliations:** 1 Department of Experimental Medicine, University of Campania “Luigi Vanvitelli”, 80138 Naples, Italy; 2 Department of Psychology, University of Campania “Luigi Vanvitelli”, 81100 Caserta, Italy; 3 Department of Clinical and Experimental Medicine, University of Foggia, 71100 Foggia, Italy; 4 Department of Movement Sciences and Wellbeing, University of Naples “Parthenope”, 80133 Naples, Italy; 5 Neurological Unit, CTO Hospital, AORN “Ospedali dei Colli”, 80131 Naples, Italy

**Keywords:** adolescent obesity, body image, body schema, depersonalization, discriminant analysis

## Abstract

Background: The relationship binding body weight to psychological well-being is unclear. The present study aims at identifying the contribution, and specificity, of some dimensions (i.e., eating-related symptoms, body image disorders, eating habits, personality traits, and emotional difficulties) characterizing the psychological profile of obese adolescents (749 participants, 325 females; 58.3% normal-weight, 29.9% overweight, and 11.7% obese; mean age = 16.05, SD = 0.82). Methods: By introducing the scores obtained by standardized self-report tools into a generalized linear model, a factorial reduction design was used to detect the best fitting discriminant functions and the principal components explaining the higher proportion of the variance. Results: We found two discriminant functions correctly classifying 87.1% of normal-weight, 57.2% of overweight, and 68.2% of obese adolescents. Furthermore, two independent factors, explaining 69.68% of the total variance, emerged. Conclusions: The first factor, “Body Image Concerns”, included the drive for thinness, body dissatisfaction, and interpersonal distrust. The second factor, “Selective Depersonalization”, included a trend toward depersonalization and dissatisfaction with the torso. The neurophysiological implications of our findings will be discussed.

## 1. Introduction

Obesity has approximately tripled in recent decades in both developed and developing countries, and the World Health Organization has formally recognized it as a global epidemic [1]. Weight gain is the most common nutritional disease in young individuals [2,3,4] and obesity at a young age is considered the main predictor of adult obesity [5,6].

It is well known that weight gain and obesity represent multifactorial conditions involving several interactions between genetic, physiologic, psychological, and social alterations [7,8,9,10,11]. However, although a negative association between obesity and physical health is widely acknowledged [12,13], the relationship binding body weight to psychological well-being is unclear. It has been hypothesized that behavioral and psychological factors could play a key role in modulating this relationship [14].

Early generations of studies focused on the link binding adolescent obesity to psychiatric disorders (e.g., affective, anxiety, somatoform disorders); however, the available data on this relationship are unclear [15,16,17,18,19,20,21,22,23,24,25,26,27,28,29,30,31]. Indeed, previous studies exploring whether obesity precedes mental disorders or whether psychological impairments boost weight gain provided inconclusive results [19]. Therefore, the link between weight gain and mental disorders does not appear to be unidirectional, and obesity was not included in the taxonomy of the latest revision of the Diagnostic and Statistical Manual of Mental Disorders (DSM-5) [32].

Later generations of studies have instead focused on the role exerted by more targeted psychological domains. In particular, self-esteem, body image perception, emotion regulation, and personality traits have been the most explored domains.

As for self-esteem, it seems to play a modulatory role in the relationship between body weight and depression [33,34]. However, only a low percentage of obese children exhibit low self-esteem coupled with depressive symptoms [35,36]. Furthermore, it has been shown that self-esteem scores in overweight and obese children generally fall within the normal range, even in clinical samples [33,37], and self-perceived body weight appears to be more closely related to reduced self-esteem rather than actual body weight [38].

Cross-sectional data have shown that body dissatisfaction in children and adolescents is a significant predictor of weight and eating disorders [39,40,41,42,43,44]. Body image is a multidimensional construct underlying how individuals perceive, think, and feel about their bodies [45]. It is usually assessed along a continuum ranging from unhealthy body perceptions (inaccurate perceptions and major negative qualities) to healthy body perceptions (accurate perceptions and predominantly positive attributes) [45,46,47]. Several studies reported that adolescent obesity is associated with increased body dissatisfaction [48], in combination with unhealthy body image and weight-related concerns [49,50,51]; it has also been found to strongly predict body dissatisfaction in adulthood [52,53,54,55,56,57,58,59].

Another line of research focused on the emotional triggers anticipating dysfunctional eating behaviors. Three main psychological-driven eating styles are related to the subjective ability to regulate emotions: emotional eating, external eating, and restrained eating [60,61,62]. Emotional eating explains overeating behaviors as a reaction to negative emotions (e.g., anxiety, depression, disappointment, loneliness) [63]. It is a maladaptive coping mechanism providing an instant reward to mitigate emotional dysregulation [64,65]. This construct is supported by physiological evidence indicating a relationship between obesity and the hypothalamic–pituitary axis (HPA) stress response [66,67,68], which would increase the ingestion of “comfort food” [69,70]. However, studies investigating the association between emotional eating and adolescent obesity have provided mixed results, highlighting positive association [71,72,73,74,75], negative association [76,77], and also no association [78,79,80]. The external eating construct [81] suggests that obese people are more sensitive to external food stimuli such as sight, smell, and taste [82,83]. However, it has been shown that external eating may also occur independently of food characteristics [80]. Finally, restrained eating implies greater efforts to limit food intake for controlling body weight [84,85]. However, it has been reported that a failure to plan and regulate food intake may induce, rather than prevent, overeating [86,87,88]. Indeed, restrictive habits may increase overeating risk as a result of excessive caloric deprivation, with cognitive control that may break down in fatigue or stress conditions [89]. Moreover, restrictive diets could be linked to both obesity and eating disorders [89,90], with episodes of binge eating often preceding prolonged periods of fasting [90].

Last but not least, some personality traits have been associated with maladaptive eating behaviors and obesity in both childhood and adolescence [91]. By definition, personality refers to a constellation of relatively stable traits underlying the individual’s tendency to think, act, and feel in a certain way [92]. Personality is related to unhealthy behaviors [93,94] such as high-calorie intake and a sedentary lifestyle [95,96,97] and it may also affect weight gain through psychological mechanisms interacting with the ability to cope with stress [98]. According to Cloninger’s psychobiological model, obese children showed lower persistence, higher novelty seeking [99], and more impulsivity compared with normal-weight subjects [71,100]. However, many of these findings arise from cross-sectional studies involving targeted clinical populations. Conversely, longitudinal studies on personality and weight gain at a young age are not extensive and provide conflicting results. Obese individuals might exhibit overeating behaviors even independently of a personological/emotional configuration [101].

In short, previous research has failed to detect a clear pattern of psychological functioning characterizing adolescent obesity [102]. This could stem from a difficulty in appreciating the extent and variability of psychological distress in the obese population. Accordingly, to date, the main challenge is to identify, among the obese subjects, which of these manifest certain psychological characteristics, and how they exhibit discomfort.

Given the progressive growth of overweight/obesity prevalence in the last decades, the relationship between obesity and behavioral/psychological dimensions—and how this affects weight rise—needs to be further explored. In particular, it may be useful to address the obesity issue in factorial terms instead of exploring selective domains. This might flatten individual variabilities likely associated with the heterogeneity of results available in the literature. Therefore, providing a multifactorial model of the psychological functioning of obese adolescents could, on the one hand, increase the reliability of psychodiagnostic assessment tools and, on the other hand, help to configure tailored weight loss treatments.

In the present pilot study, we propose a multivariate analysis, based on the generalized linear model, aimed at identifying the most affected areas of the psychological and behavioral spectrum as assessed by self-report measures (i.e., eating-related symptoms, body image, eating habits, emotional regulation processes, and personality traits) in a sample of obese adolescents residing in southern Italy. In particular, our purpose was to explore the contribution, and sensitivity, of certain domains in characterizing the psychological functioning of obese adolescents. We hypothesize the existence of a linear combination of psychological factors characterizing adolescent obesity.

## 2. Materials and Methods

### 2.1. Participants

The study was carried out on 749 participants (325 girls and 424 boys), aged between 14 and 17 years old (mean age = 16.05, SD = 0.82), recruited—via convenience sampling method—from different public high schools in the Campania region. The initiative was promoted by the Department of Experimental Medicine of the University of Campania “Luigi Vanvitelli” (Italy) and conducted by the Outpatient Clinic of Dietetics, Sports Medicine, and Psychophysical Well-Being during screening days intended for adolescent students. Inclusion criteria were the following: aged between 14 and 18 years old (to satisfy the administration criteria envisaged by the tools used); absence of intellectual or linguistic deficits; absence of neuropsychiatric disorders (e.g., schizophrenia [103], TIA, stroke, head trauma, epilepsy, major depressive disorder, bipolar disorder); absence of executive deficits (Frontal Assessment Battery–15) [104]; absence of cardiocerebrovascular diseases, cancer, type I or II diabetes, non-progressive (e.g., post-traumatic) or reversible (e.g., metabolic type, by substance intoxication, by nutritional deficiencies) cognitive impairment, connective tissue diseases (e.g., systemic lupus erythematosus, Still disease), respiratory or food allergies; no history of alcohol or drugs abuse/addiction. Furthermore, according to DSM-5 [32], no participants enrolled in the study met the diagnostic criteria for feeding and eating disorders (anorexia nervosa, bulimia nervosa, binge eating disorder, avoidant/restrictive food intake disorder, pica, rumination disorder, other specified feeding or eating disorder, unspecified feeding and eating disorders).

The anthropometric measurements (i.e., weight and height) of each participant were detected and three BMI categories were constructed in line with normative data based on the Italian cross-sectional growth charts [105], where a BMI score lower than the 85th percentile was classified as normal weight, a BMI score included in the percentile range 85th–95th was classified as overweight, and a BMI score ≥ 95th percentile was classified as obese.

### 2.2. Measures

Data were collected by using the following psychometric questionnaires. These are included in the standard assessment protocol employed by the U.O.C. of Dietetics, Sports Medicine, and Psychophysical Well-Being (University of Campania “Luigi Vanvitelli”) in the outpatient clinical practice.

Eating Disorders Inventory 2 (EDI-2) [106,107]. This is a widely used self-report measure of psychological symptoms commonly associated with eating and weight disorders. It consists of 91 items organized into 11 subscales: Drive for Thinness (DT), Bulimia (BU), Body Dissatisfaction (BD), Ineffectiveness (IN), Perfectionism (P), Interpersonal Distrust (ID), Interoceptive Awareness (IA), Maturity Fears (MF), Asceticism (ASC), Impulse Regulation (IR), and Social Insecurity (SI) (Cronbach’s α = 0.78–0.84).

Body Uneasiness Test (BUT-Form A) [108]. It is a multidimensional tool (34 items) for the clinical assessment of body uneasiness: Global Severity Index (GSI), Weight Phobia (WP), Body Image Concerns (BIC), Avoidance (A), Compulsive Self-Monitoring (CSM), and Depersonalization (D) (Cronbach’s α = 0.79–0.90).

Body Satisfaction Scale (BSS) [109,110]. It is a 16-item self-report questionnaire that measures body dissatisfaction, with each item assessing satisfaction with a specific body part. Each body part is rated on a 7-point Likert scale ranging from “very satisfied” to “very dissatisfied”. The BSS total score (BSS-Total) is obtained by summing the Head (BSS-Head), Torso (BSS-Torso), and Limbs (BSS-Limbs) sub-scores (Cronbach’s α = 0.64–0.85).

Dieter’s Inventory of Eating Temptations (DIET) [111,112]. It is an 18-item inventory designed to assess behavioral competence in six types of situations related to weight control: Overeating (OE), Negative Emotions (NE), Exercise (EX), Resisting Temptation (RT), Positive Social (PS), and Food Choice (FC). The scoring provides for a Total Score (TS-DIET) and six sub-scores for each subdomain (Cronbach’s α = 0.56–0.86).

Roman Alexithymia Scale (RAS) [113]. This questionnaire consists of 27 items allowing us to measure some relevant alexithymia components: Somatic Expression of Emotions (SEE), Difficulties to Identify the Emotions (DIE), Difficulties to Communicate the Emotion (DCE), Externally Oriented Thinking (EOT), and Difficulties to be Empathetic (DE); the sum of the five dimensions produces a total score of alexithymia (Total-RAS) (Cronbach’s α = 0.78–0.81).

Adolescent Dissociative Experiences Scale (A-DES) [114,115]. It is a screening measure for dissociative experience (i.e., dissociative amnesia, absorption and imaginative involvement, depersonalization and derealization, and passive influence). For each item, respondents indicate the frequency of the pertinent experience on an 11-point scale ranging from 0 (“never”) to 10 (“always”). The total A-DES score can be obtained by averaging across item scores (Cronbach’s α = 0.91).

Temperament and Character Inventory (TCI) [116,117]. It is a 240-item self-report questionnaire constructed to assess personality traits according to Cloninger’s psychobiological model. This model takes into account personality in seven dimensions: four temperament scales, i.e., Novelty Seeking (NS), Harm Avoidance (HA), Reward Dependence (RD), and Persistence (P), and three character scales, i.e., Self-Directedness (SD), Cooperativeness (C), and Self-Transcendence (ST) (Cronbach’s α = 0.82–0.88).

The psychometric properties of all the above-mentioned tools have been extensively explored in the Italian adolescent population. These tools are considered highly reliable for the clinical evaluation of eating behavior and weight-related disorders in Italy.

### 2.3. Statistical Analysis

To determine which psychological/behavioral variables discriminated the three BMI groups (normal-weight, overweight, obese), a stepwise discriminant function analysis was performed. According to the classification matrix, the number of correctly classified cases was reported. Then, only for the obese subgroup, the variables loading the emerged discriminant function(s) were entered into a principal component analysis (PCA) with Varimax rotation to detect any independent latent factors. The number of factors to be extracted was determined following the Mineigen criterion (i.e., eigenvalues > 1) or inspecting the scree plot. Data were analyzed using IBM SPSS Statistics for Windows, version 26 (IBM Corp., Armonk, NY, USA)and JASP packages.

## 3. Results

According to *z*-scores, no univariate outliers were detected (i.e., |3|). Square root transformation (√X_i_) was performed to normalize variables in line with skewness and kurtosis parameters (i.e., if >|1|). According to the assumption criteria of the generalized linear model, each participant belonged to a single group based on her/his BMI [105] (BMI for sex and age growth charts: normal weight, overweight, and obese). Based on Mahalanobis’ distance ($D_{i}^{2}$), no multivariate outliers were detected ($D_{i}^{2}$ = 73.402, *p* > 0.001). Multivariate normality was assumed by Mardia’s coefficient ($\;\frac{\sum_{i=1}^{N}{\left(D_{i}^{2}\right)}^{2}\;}{N}\;$= 1956.91 < 2024). Multicollinearity was assessed by either tolerance (T) or variance inflation factor (VIF). Total scores of each psychodiagnostic tool were removed since they were excessively correlated with each other. Analysis of missing data showed random missingness (MCAR) that was handled through the recommended multiple imputation method.

### 3.1. Descriptive Statistics

The whole sample included 437 normal-weight (58.3%, 185 girls), 224 overweight (29.9%, 112 girls), and 88 obese (11.7%, 28 girls) participants. According to two-way chi-squared test (χ^2^), a significant association was found between sex and BMI categories [χ^2^(2) = 8.980, *p* < 0.01, φ = 0.11]. The analysis of the adjusted standardized residuals (*z*_r_) was used as post hoc analysis [118]. Results showed that the number of female participants included in the overweight subgroup was significantly larger than expected (*z*_r_ = 2.4). Furthermore, the number of females was significantly smaller than expected (*z*_r_ = −2.3) in the obese subgroup. Descriptive statistics of the BMI-ranked group were integrated by one-way analysis of variance (ANOVA, see Table 1). Two-tailed *p*-values < 0.05 were considered statistically significant. We used Benjamini and Hochberg’s method [119] to control the false discovery rate.

### 3.2. Stepwise Discriminant Analysis

All the assumption criteria of discriminant analysis were satisfied; in particular, multivariate normality, adequate size of the smallest group (i.e., n > 20), and the ratio of variables/number of subjects (37 independent variables). Additionally, G*Power 3.1.9.4 was used to perform a power analysis for determining the number of participants required. At a nominal alpha level of 0.05, power (1–β) set to 0.80, effect size (*f*^2^*_v_*) of 0.33, 3 groups and 37 variables, the required total sample size was 81.

The analysis was performed by entering the gender variable as a covariate [*F*_(1, 36)_ = 179.36, *p* = 0.002, *η*^2^ = 0.37]. The analysis identified two discriminant functions: the first function [χ²(32) = 535.24, *p* < 0.001 (Ʌ di *Wilks* 0.482)] showed the following eigenvalues: 0.763, variance explained: 81.2%, canonical correlation: 0.658. The second function [χ²(15) = 119.42, *p* < 0.001 (Ʌ di *Wilks* 0.850)] showed the following eigenvalues: 0.177, variance explained: 18.8%, canonical correlation: 0.388. The discriminant structure matrix (Table 2) shows correlations of each variable with the respective discriminant function. The Varimax rotation procedure was performed. The rotated pooled correlations indicated that EDI-2-DT (0.476), EDI-2-BD (0.433), BSS-Torso (0.317), EDI-2-ID (0.245), and BUT-D (0.211) loaded on the first function. The second function was loaded by positive scores on the DIET-RT (0.446) and RAS-DCE (0.205) as well as negative scores on the EDI-2-IN (−0.294) and BUT-CSM (−0.237).

### 3.3. Classification Results

**Figure 1:**
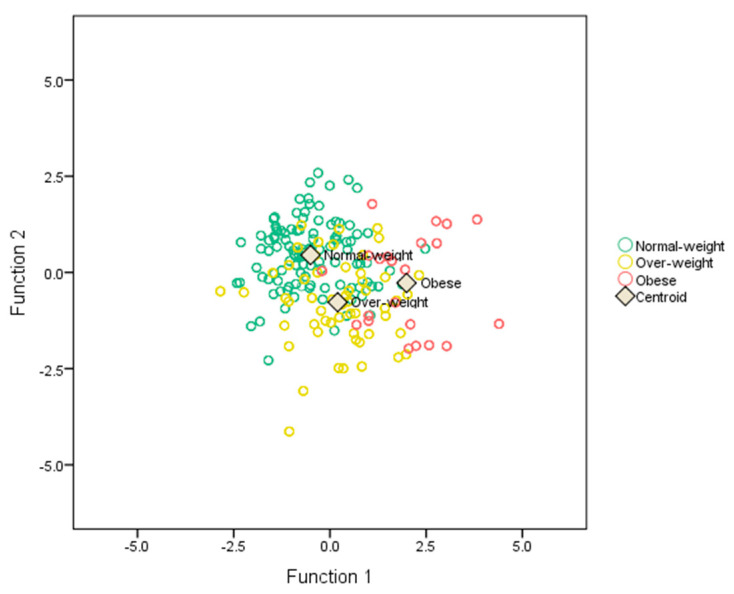
Graphical representation of each BMI-ranked group based on discriminant dimensions.

Table 3 shows the percentage of the subjects correctly vs. incorrectly classified. The extracted discriminant functions correctly classified 87.1% of normal-weight, 57.2% of overweight, and 68.2% of obese subjects. Overall, the functions correctly classified 75.9% of participants. Centroids of each group are displayed in Table 4. Normal-weight subjects were characterized by positive scores at the second function and negative scores at the first function. Overweight subjects were instead characterized by negative scores at the second function. Finally, obese subjects were characterized by positive scores in the first function and negative scores in the second function (see Figure 1).

### 3.4. Principal Components Analysis

In order to detect independent latent factors, the discriminant functions characterizing obese subjects (EDI-2-DT, EDI-2-BD, EDI-2-ID, BUT-D, and BSS-Torso) were entered into a PCA (n = 88). The rotated component matrix is reported in Table 5. The PCA revealed two factors (Bartlett’s test = 127.78, *p* < 0.001) explaining 69.68% of the total variance. The first factor, i.e., “Body Image Concerns” (eigenvalue: 2.12, variance explained: 42.48), was loaded by the EDI-2-DT, EDI-2-BD, and EDI-2-ID scores. The second factor, i.e., “Selective Depersonalization” (eigenvalue: 1.36, variance explained: 27.19), was loaded by the BUT-D and BSS-Torso scores.

## 4. Discussion

The present pilot study aimed to detect a linear combination of psychological/behavioral variables characterizing adolescent obesity. Participants completed a self-report assessment battery exploring psychological dimensions commonly related to eating and weight disorders, namely, eating-related symptoms, body image, eating habits, emotional regulation processes, and personality traits.

To determine which domain characterized each subgroup (normal-weight, overweight, and obese), a discriminant analysis was performed. The extracted functions correctly classified 75.9% of participants. In particular, 68.2% of obese subjects and 87.1% of normal-weight subjects were correctly classified. Based on the discriminant solution, two independent latent factors emerged for the obese subgroup. 

The first factor (“Body Image Concerns”) included the drive for thinness, body dissatisfaction, and interpersonal distrust. Conversely, the second factor (“Selective Depersonalization”) included depersonalization and dissatisfaction with the torso.

The “Body Image Concerns” factor was mainly explained by the drive for thinness and body dissatisfaction. By definition, the drive for thinness underlies an extreme wish to lose weight combined with an intense desire to be thinner, as well as the fear of weight gain [106]. Body dissatisfaction, instead, refers to a general discontentment with body shape, with a higher focus on the body parts most susceptible to fat deposits [106]. Finally, this factor was also explained by the interpersonal distrust reflecting a sense of alienation and reluctance to form close relationships or uncomfortableness in expressing emotions towards others [106].

Body image refers to a complex neuropsychological construct involving the individual’s perceptions, sensations, and attitudes about attractiveness and physical appearance [120,121,122,123]. In sociocultural terms, excessive thinness is constantly encouraged by media as an exclusive beauty standard [124,125], which appears to be internalized—together with body image dissatisfaction—long before puberty [126]. The internalization of a thin ideal may affect general self-determination, self-esteem, and filtering of media messages, thus increasing unhealthy eating and weight-control behaviors [127,128,129,130,131].

Furthermore, subjects with eating and weight disorders selectively focus their attention on physical appearance-related stimuli [123,132,133]. Indeed, some studies assessing attentional processing via eye movements in body exposure tasks showed that these subjects paid more attention to their self-perceived unsatisfactory body parts than to the satisfactory ones; conversely, when they looked at other people’s bodies, the opposite pattern was detected [134,135,136]. Still, in pupillometric studies using mydriasis as a physiological index of cognitive load and frontal activity [137,138], dilated pupil size, in combination with decreased blink rate (an additional index of increased attention and concentration) [139], was observed when participants allocated their attention towards their own unattractive body parts. Selective attention to certain physical features interacts with underlying knowledge structures (i.e., the body schema) filtering information, and orienting behavior [140].

Lastly, interpersonal distrust is likely the result of the idealization of the thin culture exacerbating prejudice and discrimination toward obese individuals [19]. Negative attitudes towards obese individuals might be pervasive and prejudice and discrimination may represent chronic stressors affecting the psychological well-being of obese individuals [141,142,143].

One might hypothesize that obese adolescents manifest a rumination polarized on the desire to be thin. Rumination is characterized by prolonged, repetitive, and recurrent thinking about one’s concerns and experiences [144]. According to the Goal Progress Theory [145], rumination is triggered by the perception of a discrepancy between the current state and the ideal target. In line with this claim, body dissatisfaction may trigger ruminative thoughts as a result of a dysfunctional comparison between the actual and the ideal body image.

As reported above, the second factor (“Selective depersonalization”) was explained by depersonalization and body dissatisfaction with the torso. Depersonalization is characterized by feelings of detachment and estrangement from the body. Our results seem to suggest that this phenomenon selectively involved the torso, i.e., the most dissatisfying body part.

Depersonalization is, by definition, a symptom of dissociative disorders characterized by impaired self-awareness. According to the DSM-5 criteria, it represents a persistent feeling of being detached from one’s mental processes and/or body [32], i.e., as if one is watching her/himself from the outside or as if she/he was in a dream. Depersonalization implies that mental activity, body, and the surrounding environment change in their quality, raising a feeling of unreality [146]. More specifically, disembodiment (i.e., a subdomain of depersonalization) refers to the lack of body ownership and loss of agency, namely, the feeling that actions occur automatically, irrespective of the agent’s willingness. This phenomenon ranges from an unspecific sensation of not being in the body to out-of-body experiences [147]. However, in the general population, only a few cases (1–2%) acquire clinical relevance [148]; conversely, fleeting depersonalization experiences are commonly observed, with a lifetime prevalence ranging from 26 to 74% [148,149].

It has been suggested that depersonalization provides psychological protection against acute emotional stress [150] and it is related to anxiety, stressful life events, or life-threatening situations [151]. Depersonalization may represent an inhibitory response ensuring the preservation of adaptive behaviors [147]. According to the “corticolimbic disconnection hypothesis” [147,148], fronto-limbic suppressive mechanisms would mediate an inhibitory response generating a state of emotional numbness. During depersonalization experiences, activation of the prefrontal cortex (PFC) interacts with the anterior cingulate cortex (ACC) and amygdala, generating low emotionality, attentive difficulties, autonomic mitigation, and indifference to pain [152,153,154,155]. In addition, hypoactivity of posterior parietal regions has been associated with deficits in processing and integrating somatosensory information [156,157,158,159,160,161] and low self-awareness. The somatosensory pathways are notoriously involved in both conscious perception/recognition of one’s own body (i.e., body image) [162] and in the body schema construction [163], i.e., a dynamic representation of one’s own body used to drive actions [164,165,166,167,168,169,170]. In addition, either the posterior insula or the angular gyrus also plays a significant role in integrating different input signals related to self-awareness in terms of enteroception, feelings of agency, and visceral sensations [171,172,173,174,175,176,177,178,179,180,181,182,183].

Overweight and obese adolescents are more likely to misperceive their body features, i.e., they tend to underestimate [184,185] or overestimate [178,186,187] their body weight and body parts [48,188,189,190,191], particularly when these are perceived as unattractive [135,192,193]. In the current study, selective dissatisfaction about with torso, in combination with depersonalization, was detected in obese adolescents. This result is in line with previous investigations using body size estimation-based paradigms [188,189,191].

Overall, the results of the present pilot study suggest the coexistence of two apparently opposing trends, which may instead represent two faces of the same medal. On the one hand, obese adolescents reported generalized body image dissatisfaction, accompanied by the desire to lose weight. On the other hand, they also showed a pattern of depersonalization/poor self-awareness strictly related to the most unsatisfactory body part, i.e., the torso. Further studies are needed to explore the relationship between these two clusters. In particular, future research could assess whether “Body Image Concerns” affects “Selective Depersonalization”, or vice versa. Clearly, these two factors may have a different impact on modulating behavior and interindividual differences. This may justify why some obese adolescents are motivated to undertake, and successfully conclude, weight loss programs, while other ones instead alienate from the weight problem. In this vein, integrated treatments including cognitive-behavioral and body exposure techniques [135] appear to be promising in clinical practice.

Our study presents some limitations. First, it does not allow to draw conclusions about gender differences due to the imbalanced ratio of male/female in the overweight and obese subgroups. Future studies are needed to additionally explore gender differences on the matter. However, our findings might be psychometrically transversal since we removed the effect of the sex variable. Second, our discriminant solution correctly classified 75.9% of participants by using only questionnaire data. The remaining percentage may be explained by including more objective measures of body representation (for instance, quantitative estimations of own body size, and heartbeat detection accuracy as a measure of interoceptive awareness (see [48] for additional details)). Third, psychological traits were evaluated by self-reported questionnaires which notoriously tend to overestimate the prevalence of psychopathology [194]. Fourth, results are restricted to students enrolled in screening initiatives and do not provide any information on the larger number of obese individuals not seeking treatment or seeking help in non-clinical settings. Finally, we did not take into account the role exerted by maturation processes or family history of obesity.

## 5. Conclusions

Our preliminary results showed that obesity negatively affects body perception and subjective body experience in obese adolescents. In particular, obese subjects are characterized by two relatively independent psychological factors exerting a critical influence on body representation. On the one hand, we detected a cognitive body image dissatisfaction mainly associated with an excessive desire for thinness. On the other hand, obese adolescents showed a feeling of estrangement and detachment from the body region perceived as the most unpleasant, such as the torso. In sum, the discomfort observed in both the “explicit” and “implicit” dimensions demonstrates that the representation of one’s own body plays a critical role in adolescent obesity, much more so than other variables explored.

## Figures and Tables

**Table 1:** Mean raw scores (SDs) for each scale.

	Normal-Weight (n = 437)	Overweight (n = 224)	Obese (n = 88)	*p*-Value
***Eating Disorder Inventory 2 (EDI-2)***				
Drive for Thinness—DT	3.8 (5)	8.1 (5.8)	11.6 (5.5)	<0.001 *
Bulimia—BU	2.4 (2.8)	2.7 (3.7)	3.1 (3)	0.156
Body Dissatisfaction—BD	6.4 (5.8)	12.8 (7.6)	16 (6.1)	<0.001 *
Ineffectiveness—IN	3.1 (3.4)	4.7 (4.5)	4.2 (3.9)	<0.001 *
Perfectionism—P	4.7 (3.2)	5 (3.7)	5.4 (3)	0.175
Interpersonal Distrust—ID	3.4 (2.8)	3.5 (2.8)	5 (3.6)	0.006 *
Interoceptive Awareness—IA	5.1 (5)	6 (4.9)	6.2 (6.6)	0.113
Maturity Fears—MF	6.3 (3.7)	6.9 (4.8)	7 (3.7)	0.425
Asceticism—ASC	3.3 (2.8)	4.1 (3.1)	4.6 (3.3)	0.001 *
Impulse Regulation—IR	4.3 (4.3)	5.8 (4.8)	5.9 (5.7)	<0.001 *
Social Insecurity—SI	3.4 (2.4)	4 (3.4)	4.2 (3.9)	0.048
***Body Uneasiness Test (BUT-Form A)***				
Global Severity Index—GSI	0.8 (0.7)	1.4 (1)	1.5 (0.8)	<0.001 *
Weight Phobia—*WP*	1.2 (0.8)	2 (1.2)	2 (0.9)	<0.001 *
Body Image Concerns—BIC	0.9 (0.8)	1.9 (1.3)	2.2 (1.1)	<0.001 *
Compulsive Self-Monitoring—CSM	0.9 (0.8)	1.2 (0.9)	1.2 (0.8)	<0.001 *
Avoidance—A	0.4 (0.7)	0.7 (0.8)	0.8 (0.9)	<0.001 *
Depersonalization—D	0.5 (0.7)	0.8 (0.9)	1 (0.9)	<0.001 *
***Body Satisfaction Scale (BSS)***				
BSS-Total	35.1 (12)	38.8 (9.8)	40.9 (11.6)	<0.001 *
BSS-Head	13.8 (5.1)	12.9 (4.6)	13.6 (6)	0.377
BSS-Torso	10.9 (4.3)	12.7 (3.8)	14.7 (4.2)	<0.001 *
BSS-Limbs	10.4 (4.8)	13.2 (4.8)	12.6 (4.9)	<0.001 *
***Dieter’s Inventory of Eating Temptations (DIET)***				
DIET Total Score	4.3 (1)	4.1 (0.9)	4.1 (0.9)	0.042
Overeating—OE	3.7 (1.5)	3.8 (1.5)	3.6 (1.4)	0.811
Negative Emotions—NE	4.8 (1.4)	4.7 (1.3)	4.4 (1.5)	0.233
Positive Social—PS	4.6 (1.6)	4.2 (1.3)	4.1 (1.2)	0.009 *
Food Choice—FC	3.1 (1.5)	3.0 (1.2)	2.9 (1.4)	0.810
Resisting Temptation—RT	5.1 (1.6)	4.3 (1.5)	4.6 (1.5)	<0.001 *
Exercise—EX	3.9 (1.3)	3.9 (1.2)	4.2 (1.5)	0.067
***Roman Alexithymia Scale (RAS)***				
Somatic Expression of Emotions—SEE	8.9 (2.5)	9.3 (3.0)	9.2 (3.4)	0.753
Difficulties to identify the emotions—DIE	13.2 (3.4)	14.4 (3.4)	12.8 (3.3)	<0.001 *
Difficulties to communicate the emotion—DCE	10.6 (2.9)	9.3 (2.3)	9.7 (2.3)	<0.001 *
Externally oriented thinking—EOT	10.2 (1.9)	10.3 (2.3)	10.3 (2.6)	0.841
Difficulties to be empathetic—DE	11.4 (2.3)	11.1 (2.1)	12.2 (2.7)	<0.001 *
Total score RAS	53.4 (7.1)	54.3 (8.3)	55.1 (10)	0.363
***Adolescent Dissociative Experiences Scale (A-DES)***	1.9 (1.4)	1.9 (1.3)	2.3 (1.8)	0.862
***Temperament and Character Inventory (TCI)***				
Novelty Seeking—NS	20.9 (4.7)	20.8 (5.7)	20.2 (4.5)	0.410
Harm avoidance—HA	46.5 (36.2)	52.5 (40.8)	45.4 (40.4)	0.142
Reward Dependence—RD	15.6 (3.3)	15.9 (2.9)	15.4 (3)	0.662
Persistence—P	4.5 (1.6)	4.4 (1.5)	4.7 (1.7)	0.281
Self-Directedness—SD	26.2 (6)	25.5 (7.7)	28.3 (7.3)	0.001 *
Cooperativeness—C	30.2 (5.6)	30.3 (5.2)	30.1 (5.8)	0.846
Self-Transcendence—ST	17.3 (5.3)	16 (5.6)	14.8 (5.6)	<0.001 *

Note: ANOVAs were significant (*) according to Benjamini and Hochberg’s adjusting method.

**Table 2:** Correlations between the discriminating variables and the canonical functions.

	Functions	
	1	2
EDI-2—Drive for Thinness—DT	0.476	
EDI-2—Body Dissatisfaction—BD	0.433	
BSS-Torso	0.317	
EDI-2—Interpersonal Distrust—ID	0.245	
BUT—Depersonalization—D	0.211	
DIET—Resisting Temptation—RT		0.446
EDI-2—Ineffectiveness—IN		−0.294
BUT—Compulsive Self-Monitoring—CSM		−0.237
RAS—Difficulties to communicate the emotion—DCE		0.205

**Table 3:** Classification results based on discriminant analysis.

BMI	Predicted Group Membership (%)			Total
	Normal-Weight	Overweight	Obese	
Normal-weight	**87.1**	10.1	2.8	100.0
Overweight	37.4	**57.2**	5.4	100.0
Obese	18.2	13.6	**68.2**	100.0

**Table 4:** Functions at group centroids.

	Functions	
	1	2
Normal-weight	−0.505	0.452
Overweight	0.199	−0.775
Obese	0.992	−0.274

**Table 5:** Principal component matrix.

	Component	
	1	2
EDI-2—Drive for Thinness—DT	0.899	
EDI-2—Body Dissatisfaction—BD	0.824	
EDI-2—Interpersonal Distrust—ID	0.474	
BUT—Depersonalization—D		0.904
BSS-Torso		0.889

Rotation method: Varimax with Kaiser normalization.

## Data Availability

The data presented in this study are available on request from the corresponding author.
